# The Effect of Corporate Social Responsibility Compatibility and Authenticity on Brand Trust and Corporate Sustainability Management: For Korean Cosmetics Companies

**DOI:** 10.3389/fpsyg.2022.895823

**Published:** 2022-05-12

**Authors:** Su-Hee Lee, Gap-Yeon Jeong

**Affiliations:** ^1^Department of Beauty Design, Catholic Kwandong University, Gangneung-si, South Korea; ^2^Department of International Trade, Andong National University, Andong, South Korea

**Keywords:** corporate social responsibility, brand trust, corporate sustainability management, Korean cosmetics company, Korean cosmetics market

## Abstract

The purpose of this study is to examine whether corporate social responsibility (CSR) activities perceived by consumers affect brand trust and corporate sustainability management (CSM). In other words, this study tried to examine whether the compatibility and authenticity of CSR influences brand trust, thereby affecting CSM including economic viability, environmental soundness, and social responsibility. To measure this, an empirical analysis was conducted on 479 consumers who had experience purchasing products from cosmetic companies that are carrying out CSR. As a result of the analysis, it was found that the compatibility and authenticity of CSR have a positive effect on brand trust. Also, it was found that brand trust had a positive effect on social responsibility among the sub-concepts of CSM, but did not affect economic viability and environmental soundness. The results of this study are expected to provide strategic implications for social responsibility performance and brand trust building necessary for cosmetics companies to grow continuously.

## Introduction

As companies’ influence on society increases, corporate social responsibility (CSR) has become an essential factor that companies must choose to realize sustainable management. CSR is a process of fulfilling economic and environmental responsibilities required by society to realize human values as well as maximize profits (Clark, 2000). CSR can have a direct effect on the formation of positive attitudes toward companies and products by consumers, thereby enhancing customer loyalty and business performance (Sen et al., 2006). Therefore, it is necessary for companies to consider CSR as a key means to enable corporate sustainability management and to strengthen strategic approaches using it (Konrad et al., 2006).

As sustainable development for mankind and social awareness become more important, corporate sustainability is emerging as an important issue (Lloret, 2016). In addition, as problems such as global economic crisis, social conflict, climate change and environmental pollution continued, “sustainability” became the most fundamental goal in corporate management (Benessia and Funtowicz, 2015). Unilever (2017) reported that the sustainable market is worth about 3,200 trillion won, of which 125 trillion won is a potential market that has not yet been developed. Accordingly, as a part of sustainable management, companies have come up with various strategies to increase social interest in eco-friendliness.

Corporate Sustainability Management (CSM) refers to management activities that do not harm current and future generations and secure corporate economic outcomes as well as striving for ethical, environmental, and socially sustainable development (Benessia and Funtowicz, 2015). CSM is based on environmental responsibilities, including producing eco-friendly products and participating in environmental movements, and social responsibilities, including creating jobs and improving human resources with the profits generated by the company (Elkington, 1997). These activities yield ethical management, innovative management, social responsibility management, environmental management, and creative management within the company. Outside the company, they enhance consumers’ knowledge or perceived value of the company and positively affect trust, corporate image, and customer loyalty, thereby strengthening a company’s competitive edge (Forehand and Grier, 2003; Luo and Bhattacharya, 2006).

Amid the changing business environment, companies are carrying out CSR that create economic and social values for sustainable growth (Matten and Moon, 2008). However, CSR was perceived by consumers as an activity for business to generate profits, resulting in negative attitudes of consumers (Webb and Mohr, 1998). Therefore, the compatibility and authenticity of CSR that can attract consumers’ participation and interest is important (Becker-Olsen and Hill, 2006). These concepts have an important effect on consumers’ assessment of CSR as well as attitudes toward companies (Sen et al., 2006).

Corporate social responsibility compatibility refers to the degree to which consumers recognize whether there is a correlation between a company’s characteristics, such as brand image and products, and CSR (Gupta and Pirsch, 2006). Should there be a high compatibility between the company’s features and CSR, consumers can establish trust in the company, ultimately forming commitment and loyalty between the company and the consumer (Becker-Olsen and Hill, 2006; Koschate-Fischer et al., 2012). In addition, high compatibility creates a positive corporate and brand image (O’Connor and Meister, 2008).

Corporate social responsibility authenticity means that a company carries it out with pure intentions (Price et al., 1995). Even if the company and CSR are compatible, the company cannot achieve any outcome should consumers recognize CSR as profit-seeking, hypocritical activities (Price et al., 1995). Sincere CSR with authenticity results in favorable responses from consumers and has a positive effect on consumers’ trust in and attitude toward the company, as well as their purchase intention (Alhouti et al., 2016). Therefore, should consumers feel that CSR is authentic, they will positively evaluate corporate activities and recognize them as ethical companies, establishing trust in the company and brand (Sen and Bhattacharya, 2001; Becker-Olsen and Hill, 2006).

Brand trust refers to the belief that a brand will fulfill consumers’ best interests for goals or values shared by the consumers (Chaudhuri and Holbrook, 2001). Since brand trust affects the relationship between consumers and the brand, it is one of the factors that promote the continuous growth of a company (Erdem and Swait, 2004; Sichtmann, 2007). Therefore, companies must build brand trust in order to form a positive relationship with consumers. In other words, should a company carry out compatible and genuine CSR activities, it can establish a successful relationship with consumers, thereby raising brand trust.

This study aims to examine whether the CSR of cosmetics companies perceived by consumers affect brand trust and CSM. In other words, this study aims to examine whether the compatibility and authenticity of CSR affects brand trust, and whether the aforementioned brand trust affects CSM, which is comprised of economic viability, environmental soundness, and social responsibility. Cosmetics companies were selected as the subject in this study because they used chemical stock and plastic containers in the past, and as these materials have become one of the main causes of environmental destruction, cosmetics companies have started to invest in and research sustainable development, including eco-friendly packaging, reduction of waste and carbon emissions, and research on alternative substances, to protect the natural environment (Bom et al., 2020). For example, The Body Shop, a Korean cosmetics company, promotes anti-animal testing, promoting self-respect, protecting human rights, supporting fair trade, and protecting the global environment as sustainable management. Amore Pacific also promotes a sustainable lifestyle, grows together with economic and social communities, and contributes to a circular economy for future generations (Lassk, 2019). The outcome of this study is expected to provide strategic implications for establishing the CSR and brand trust necessary for the continuous growth of cosmetics companies.

## Theoretical Background

### Corporate Social Responsibility Compatibility and Authenticity

Corporate social responsibility (hereinafter referred to as CSR), a term first used by Bowen in the 1950s, refers to pure supporting activities that do not have commercial purposes and it is the duty of entrepreneurs to make policies and decisions that fit the purpose and value of society and are considered desirable (Bowen, 1953). Since then, studies on CSR have presented ethical as well as social aspects and defined social responsibility more broadly and comprehensively. Carroll (1983) stated that CSR includes economic, legal, ethical, and benevolent expectations that society has for a company. In other words, CSR fulfills the economic and environmental responsibilities required by society by prioritizing profit maximization along with human value realization (Clark, 2000). CSR is carried out in various ways, including research on its purpose and research on factors that affect performance (Joyner and Payne, 2002). Recently, studies have been conducted to find out whether CSR has a direct effect on consumers’ attitude and behaviors toward companies (Backhaus et al., 2002). CSR establishes a positive image of a company, and as it increases consumer loyalty by inducing a caring reputation, CSR promotes the improvement of corporate financial performance (Doh et al., 2010).

Compatibility and authenticity play an important role in consumer behavior and attitudes and have the greatest influence on CSR (Beckman et al., 2009). CSR compatibility refers to the degree to which consumers recognize whether there is a correlation between a company’s characteristics, such as brand image and products, and CSR (Gupta and Pirsch, 2006). CSR tailored to the characteristics of a company can enhance the effectiveness of CSR by making it easier for consumers to understand the company and its products and quickly recognize and accept CSR (Sen et al., 2006). In other words, the higher the CSR compatibility, the more positive attitudes and trust consumers have toward corporate reputation and image (O’Connor and Meister, 2008). In this way, CSR compatibility is an important factor in determining the effectiveness of CSR (Becker-Olsen and Hill, 2006).

Corporate social responsibility authenticity refers to the degree to which a company is carrying out CSR with pure intentions (Price et al., 1995). Consumers may suspect that companies engage in CSR as a means of pursing profits or overcoming crises (Beckman et al., 2009). In other words, the effectiveness of CSR may vary depending on how consumers perceive the authenticity of CSR (Forehand and Grier, 2003). When consumers infer that a company carries out CSR to pursue economic outcomes, they realize authenticity is low. When consumers infer that a company carries out CSR out of pure acceptance of social needs, they may consider that the company is authentic and think highly of the company (Becker-Olsen and Hill, 2006). Therefore, CSR authenticity is an important factor influencing the outcome of CSR, and companies must carry out authentic CSR activities (Becker-Olsen and Hill, 2006; Beckman et al., 2009; Alhouti et al., 2016). Compatibility and authenticity, which are elements for evaluation of CSR, are seen as essential factors in achieving a competitive edge (Gilmore and Pine, 2007).

### Brand Trust

Brand trust refers to consumers’ belief that the brand will fulfill the best interests for the consumers in order to achieve the goals or values shared by the company and consumers, and such trust will have a positive effect on consumers’ decision-making processes (Doney and Cannon, 1997; Chaudhuri and Holbrook, 2001). Moreover, brand trust is achieved from the relationship between the consumer and the brand and established after the consumer experiences a specific brand (Del Vecchio, 2000). Therefore, brand trust is an important factor in promoting a company’s long-term growth as it affects the relationship between consumers and a company (Erdem and Swait, 2004; Sichtmann, 2007).

According to previous studies on brand trust, Morgan and Hunt (1994) argued that the establishment of a successful relationship between a brand and consumers would bring a positive effect on consumer loyalty and commitment to the brand. Doney and Cannon (1997) stated that trust between the consumers and brand plays an important role in situations in which a consumer has to make a purchasing decision before experiencing a specific brand. Lafferty and Goldsmith (1999) argued that CSR can create a positive corporate image and raise brand trust. In other words, the establishment of brand trust promotes customers’ positive words and purchase intentions, thereby promoting the company’s long-term performance (Kotler and Armstrong, 2013). Brand trust is a motivational factor promoting consumer consumption and plays an important role in building a positive relationship between a brand and consumers (Gwinner et al., 1998). Therefore, brand trust is a future behavior subscale expected of consumers in a competitive marking environment and an essential factor that companies must manage for long-term performance (Vogel et al., 2008).

### Corporate Sustainability Management

The concept of sustainability began to form as it was suggested that human growth would reach its limit due to environmental pollution and resource depletion caused by economic development. Sustainable development was first proposed by the 1987 Brundtland Report from the World Commission on Environment and Development (WCED) (Carroll and Shabana, 2010). Sustainable development satisfies the needs and desires of the present generation while presenting eco-friendly development without hindering the ability of subsequent generations to meet their needs (WCED, 1987). Such sustainable development and management activities that fulfill corporate social responsibility can be referred to as CSM. In other words, CSM includes environmental and ethical responsibilities as well as economic, social, and legal responsibilities required by society (Schaltegger and Hörisch, 2017). Therefore, rather than pursuing profit, economic responsibility, and legal responsibility, CSM accompanies economic, social, environmental, and ethical management, serving as a positive role in the community, country, and world society beyond internal and external stakeholders (Elkington, 1997). Although CSM is perceived as a similar meaning to CSR, there is a clear difference. While CSM focuses on the continuous development and growth of companies, CSR focuses on issues for social development (Van Marrewijk and Werre, 2003).

Triple Bottom Line (TBL) is the most common term used in CSM. TBL presented by Elkington (1997) refers to a situation in which companies are economically viable and environmentally sound and strive to sustain CSR. It consists of economic viability, environmental soundness, and social responsibility (Craig and Rogers, 2008). Among the factors of TBL, economic viability is essential to secure, maintain, and efficiently distribute benefits with the stakeholders in the long run (Amalric and Hauser, 2005). Environmental soundness refers to the management of the environment within the boundaries of the law, production of eco-friendly products to enhance corporate reputation, and participation in environmental movements and campaigns to raise the standard of life for all members of society (Elkington, 1997). Social responsibility, essential for companies to practice sustainable management, refers to the act of returning a portion of the profits produced by a company to society. Not only that, it includes job creation and the improvement of human resource related infrastructure (Becker-Olsen and Hill, 2006). Social responsibility is the effort of a company to create new social capital to achieve social goals. Social capital is a concept that a company builds trust with its stakeholders. In order for companies to be sustainable, social capital must be promoted (Becker-Olsen and Hill, 2006). In other words, all activities regarding management must maintain and manage social systems and ecosystems by harmonizing with economic, environmental, and social responsibilities, and TBL is a practical tool for practicing sustainable management and is being utilized in corporate management by means of basic and universal methods (Carroll and Shabana, 2010).

According to studies on CSM, King and Lenox (2001) reported that practicing sustainable management has a positive impact on the values and financial outcome of a company. Moreover, Craig and Rogers (2008) summarized the concept of CSM and reported on the relationship between long-term economic viability and environmental, social, and economic performance. Porter and Kramer (2011) argued that a company can gain its competitive edge by securing more valuable resources than its competitors and practicing CSR; therefore, it can facilitate CSM.

## Research Method

### Research Model

**FIGURE 1:**
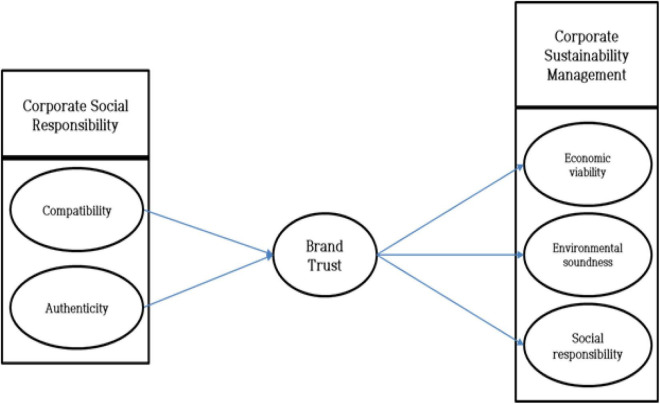
Research model.

When consumers perceive that CSR is authentic and suitable for the company’s products, they feel trust in the company’s brand (Becker-Olsen and Hill, 2006; Sen et al., 2006). In other words, consumers can perceive a company as ethical if its CSR compatibility and authenticity are high, thereby establishing brand trust and satisfaction (Becker-Olsen and Hill, 2006; Alhouti et al., 2016). In addition, consumer trust in the brand improves the company’s sustainability management because it makes consumers trust the company and continue to purchase the product (Hon and Grunig, 1999). Therefore, this study proposed a research model as shown in Figure 1 to determine whether CSR compatibility and authenticity affect brand trust, and brand trust affects CSM, which includes economic viability, environmental soundness, and social responsibility.

### Hypotheses

#### Corporate Social Responsibility and Brand Trust

Consumers “awareness of CSR can have an important impact on consumers” attitudes to the brand. In particular, it is important whether the CSR fits well with the company’s product and whether the CSR is genuine (Beckman et al., 2009). CSR compatibility refers to the degree to which consumers recognize whether there is an association between a company’s characteristics, such as brand image and products, and CSR (Gupta and Pirsch, 2006). CSR that is highly compatible with corporate brands can result in positive consumer attitudes (Forehand and Grier, 2003). According to Rifon et al. (2004), if CSR and corporate characteristics are highly compatible, consumers establish trust in the company, hence, it can secure a positive corporate image. Becker-Olsen and Hill (2006) argued that a high CSR compatibility yields a strong association between the company and CSR, and as a result, consumers positively evaluate the brand.

Corporate social responsibility authenticity refers to the degree to which a company is carrying out CSR with pure intentions (Price et al., 1995). Brown and Dacin (1997) stated that authentic CSR has a positive effect on consumers’ positive attitudes toward the products and services. Schallehn et al. (2014) argued that consumers positively evaluate corporate activities and perceive the company as ethical when they consider CSR as a sincere act, thereby establishing trust in the company and brand. On that account, the following hypotheses were established.

H1: *CSR compatibility will have a positive effect on brand trust.*

H2: *CSR authenticity will have a positive effect on brand trust.*

#### Brand Trust and Corporate Sustainability Management

Brand trust refers to the belief that a brand will fulfill the consumers’ best interests for the goals or values shared by the consumers (Chaudhuri and Holbrook, 2001). Since brand trust affects the relationship between a company and consumers, it is an important factor in maintaining and strengthening long-term transactions between them, thereby inducing sustainable growth of the company (Erdem and Swait, 2004; Sichtmann, 2007).

When consumers perceive that the trade of products or services is honest and fair and establish trust, they will be fonder of the brand, forming a long-term relationship (Ganesan, 1994; Garbarino and Johnson, 1999). According to Ganesan (1994), when consumers establish trust by observing and anticipating a company, the quality of mutual relationship improves, and the relationship can be prolonged. Erdem and Swait (2004) argued that brand trust is built in the faith that the brand will fulfill its promise in the long run, and consequently, consumers will more likely choose the brand while maintaining a long-term relationship (Doney and Cannon, 1997; Chaudhuri and Holbrook, 2001). Therefore, the following hypotheses were established.

H3: *Brand trust will have a positive effect on CSM.*

H3-1: *Brand trust will have a positive effect on economic viability.*

H3-2: *Brand trust will have a positive effect on environmental soundness.*

H3-3: *Brand trust will have a positive effect on social responsibility.*

### Measurement of Variables

The variables used in this study were modified and supplemented from the questionnaires verified in previous studies to suit the purpose of this study. All measurement items were evaluated on a Five-Point Likert scale with “Strongly disagree” as 1 point, “Neutral” as 3 points, and “Strongly agree” as 5 points (See Appendix).

This study defines CSR compatibility as the degree to which consumers recognize whether there is an association between a company’s characteristics, such as brand image and products, and CSR. To measure this factor, a total of three items were used based on the studies of Becker-Olsen and Hill (2006) and O’Connor and Meister (2008). CSR authenticity is defined as the extent to which consumers perceive that a company is carrying out CSR with pure intentions, and to measure this, a total of three items were established based on a study by Alhouti et al. (2016).

Brand trust is defined as the belief that a brand will fulfill the consumers’ best interests for the goals or values shared by the consumers, and to measure this, a total of five questions were used based on the studies of Keller and Aaker (1992) and Mayer et al. (1995).

Corporate sustainability management is defined as the degree to which consumers perceive that a company is engaged in management activities not only for its economic outcome, but also for continuous economic and social developments. Moreover, three sub-concepts – economic viability, environmental soundness, and social responsibility – were established based on Elkington’s (1997) concept of Triple Bottom Line. Economic viability is defined as the degree to which a company engages in management activities to secure economic profits and maintain them in the long run. A total of four questions were composed by referring to a study done by Amalric and Hauser (2005). Environmental soundness is defined as the degree to which a company manages the environment and produces eco-friendly products to increase the standard of lives of all members of society, and a total of four questions were composed by referring to a study done by Chen et al. (2006). Social responsibility is defined as the degree of management activities that return a portion of the profits to society, and four questions were composed based on a study by Kotler and Lee (2005).

### Research Data and Analysis Method

The questionnaire consisted of three questions on CSR compatibility, three on authenticity, five on brand trust, and 12 on CSM. A preliminary questionnaire was conducted on 20 adult men and women who have used products from Amore Pacific and The Body Shop, which both carry out CSR. After that, the survey contents were revised and supplemented. Conducted on adult men and women who have used products from Amore Pacific and The Body Shop, this questionnaire was conducted for almost 3 months from March 4 to May 30, 2021. A total of 500 copies of the questionnaire were distributed, of which a total of 479 copies were used for empirical analysis, excluding 21 copies with insincere responses. These pieces of data were empirically analyzed *via* SPSS WINDOW 21.0 and AMOS 19.0 statistical analysis programs.

In this research, we conducted structural equation model analysis instead of regression analysis which had been used. Because structural equation model that confirms structural relation among multiple variables is more suitable than regression analysis that is limited to research casual relations among multiple valuables. Also, regression analysis can analyze using error of variable’s multiple factor scale value, on the other hand, if measurement error occurs, structural equation model analysis’s fit of model go down. Therefore, structural equation model analysis is more suitable than regression analysis to examine exactly relationship with multiple variables.

## Empirical Analysis

### Demographic Characteristics of Respondents

The total effective sample of this study is 479 people, and the demographic characteristics of the respondents are shown in Table 1.

**TABLE 1:** Demographic characteristics of respondents.

	Content	Number of respondents	%
Gender	Male	225	47.0
	Female	254	53.0
Age	Under 25	77	16.1
	26–30	79	16.5
	31–35	144	30.1
	36–40	98	20.5
	Over 41	81	16.9
Marriage	Single	291	60.8
	Married	188	39.2
Education	High School Graduate	58	12.1
	Junior College Graduate	117	24.4
	University Graduate	220	45.9
	Graduate School	84	17.5
Job	Student	34	7.1
	Housewife	65	13.5
	White Collar	137	28.5
	Official	128	26.6
	Specialized work	73	15.2
	Others	42	8.7
Where to buy cosmetics	Online	114	23.8
	Department store	125	26.1
	Duty free shop	84	17.5
	Specialty store	118	24.6
	Others	38	7.9
Average monthly cosmetic usage amount	Less than 50,000 Won	65	13.6
	50,000–100,000 Won	125	26.1
	100,000–200,000 Won	154	32.2
	200,000–300,000 Won	104	21.7
	300,000 Won or more	31	6.5
Average monthly income	Less than 1 million won	27	5.6
	1–2 Million won	61	12.7
	2–3 Million won	105	21.9
	3–4 Million won	157	32.8
	4–5 Million won	91	19.0
	5 Million won or more	38	7.9
Total		479	100

### Reliability and Validity

In this study, confirmatory factor analysis was performed to examine reliability and validity. First, reliability verification was analyzed using Cronbach’s α, an index that confirms the internal consistency of measurement items. As a result of the analysis, Cronbach’s α of the measurement items was higher than the standard value of 0.6, so it was judged that the reliability of the measurement items of this study was secured (Anderson and Gerbing, 1988). The reliability results are shown in Table 2.

**TABLE 2:** Reliability analysis.

Construct		Number of first items	Number of final items	Cronbach’s α
CSR	Compatibility	3	3	0.928
	Authenticity	3	3	0.887
Brand trust		5	5	0.858
CSM	Economic viability	4	4	0.875
	Environmental soundness	4	4	0.856
	Social responsibility	4	4	0.924

Second, we tried to examine the validity of the variables in this study, especially the convergent validity and discriminant validity. Convergence validity can be judged when the construct reliability (CR) value is 0.7 or higher and the average variance extracted (AVE) value is 0.5 or higher. And discriminant validity can be checked when the AVE values of the variables in this study are greater than the squared values of the correlation coefficients (Hair et al., 2005). According to the analysis result (refer to Table 3), both CR and AVE values of the variables were above the standard values, so it can be judged that the variable items have convergence validity. Among the variables, brand trust (0.514) with the smallest AVE value was larger than the squared value (0.190) of compatibility and authenticity (0.436) with the largest correlation value, indicating that there is no problem with discriminant validity (refer to Tables 3, 4).

**TABLE 3:** Confirmation factor analysis.

Construct		Factor	Standard estimate	*t*-Value	CR	AVE
CSR	Compatibility	CP1	0.844	–	0.876	0.526
		CP2	0.785	10.288		
		CP3	0.754	10.242		
	Authenticity	AT1	0.856	–	0.885	0.549
		AT2	0.853	10.935		
		AT3	0.797	10.386		
Brand trust		BT1	0.847	–	0.816	0.514
		BT2	0.804	10.513		
		BT3	0.820	10.642		
		BT4	0.796	10.292		
		BT5	0.784	10.287		
CSM	Economic viability	EV1	0.842	–	0.913	0.611
		EV2	0.811	10.522		
		EV3	0.793	10.290		
		EV4	0.780	10.283		
	Environmental soundness	ES1	0.824	–	0.894	0.563
		ES2	0.803	10.512		
		ES3	0.790	10.289		
		ES4	0.742	10.225		
	Social responsibility	SR1	0.823	–	0.915	0.613
		SR2	0.815	10.524		
		SR3	0.814	10.523		
		SR4	0.785	10.288		

*χ^2^ = 132.384, df = 136, p = 0.000, GFI = 0.913, CFI = 0.927, NFI = 0.924, RMR = 0.064, RMSEA = 0.059.*

**TABLE 4:** Correlation analysis.

	CP	AT	BT	EV	ES	SR
CP	1					
AT	0.436**	1				
BT	0.223**	0.189**	1			
EV	0.308**	0.398**	0.211**	1		
ES	0.304**	0.369**	0.188**	0.307**	1	
SR	0.193**	0.301**	0.296**	0.230**	0.238**	1
Average	3.7124	3.4398	3.4497	3.5269	3.7471	3.5688
Standard Deviation	0.6708	0.7831	0.6892	0.6789	0.6833	0.6793

**p < 0.01.

As a result of examining the fit indices of the measurement model according to the confirmatory factor analysis (refer to Table 3), it was confirmed that various suitability indices were higher than the standard value (χ^2^ = 132.384, df = 136, *p* = 0.000, GFI = 0.913, CFI = 0.927, NFI = 0.924, RMR = 0.064, RMSEA = 0.059). The results of the confirmatory factor analysis are shown in Table 3.

### Correlation Analysis

Correlation analysis was performed to confirm the problem of multi-collinearity between variables. The correlation between most variables was statistically significant at 0.01. Among the correlations between variables, the relationship between suitability and authenticity showed the highest value at the significance level of 0.01–0.436, but the correlation between other variables was 0.5 or less. Therefore, it was judged that there was no problem of multi-collinearity among the variables in this study (Hair et al., 2005). The results of the correlation analysis are shown in Table 4.

### Hypothesis Test

The hypothesis was verified by confirming the significance of the path coefficient of the structural equation model. The suitability of this research model was examined prior to hypothesis testing, and as a result, it can be judged that the research model is suitable for hypothesis testing (χ^2^ = 135.173, df = 132, *p* = 0.000, GFI = 0.917, CFI = 0.928, NFI = 0.926, RFI = 0.938, IFI = 0.909, TLI = 0.912). Hypothesis testing can generally be judged according to the criterion of *t*-value according to the significance level (*p* < 0.05: *t*-value 1.96–2.58, *p* < 0.01: *t*-value over 2.58). The hypothesis test results are shown in Table 5.

**TABLE 5:** Hypothesis test.

	Hypothesis	Path coefficient	Standardized path coefficient	*t*-Value	*p*-Value	Result
H1-1	Compatibility → Brand trust	0.191	0.202	4.495	0.000	Accept
H1-2	Authenticity → Brand trust	0.279	0.244	5.490	0.000	Accept
H2-1	Brand trust → Economic viability	0.011	0.055	0.318	0.751	Reject
H2-2	Brand trust → Environmental soundness	0.006	0.007	0.159	0.874	Reject
H2-3	Brand trust → Social responsibility	0.154	0.175	3.893	0.000	Accept

## Conclusion and Discussion

The purpose of this study was to determine whether the CSR compatibility and authenticity of cosmetics companies affect CSM, which consists of economic viability, environmental soundness, and social responsibility, through brand trust. The results of the empirical analysis confirmed that the compatibility and authenticity of CSR did have a positive effect on brand trust. Additionally, brand trust was confirmed to have a positive effect on social responsibility, but not on economic viability and environmental soundness. The specifics and implications of the results of this study are as follows.

First of all, it was confirmed that CSR compatibility and authenticity of cosmetics companies have a positive effect on brand trust. This outcome indicates that consumers are very interested in the nature of CR, compatibility with corporate culture, and business purpose, and that compatibility and authenticity, which are the motivations for CSR, are significant in building brand trust. No matter how good-natured CSR is, if it does not match the company’s characteristics, consumer trust will inevitably fall. Therefore, it is important to determine whether the type of CSR carried out by cosmetics companies is related to the company and plan the CSR strategies and directions. In other words, it is crucial to find issues that can represent the core competence of cosmetics companies rather than social issues, such as environmental problems and support for the poor. In addition, it is important to find the targets for CSR, which match the company’s image, and clearly display the company’s characteristics, drawing responses from consumers. Furthermore, cosmetics companies need a strategy that provides consumers the perception that their CSR is authentic by prioritizing the social good over individual economic benefits. Therefore, the company has to actively provide information, such as outcome and ripple effect of CSR, using corporate advertisements and communication tools so that consumers can establish trust in the company as well as the brand.

Second, brand trust was confirmed to have a positive effect on the social responsibility of CSM. This outcome shows that consumers’ trust in the brand is an important factor in establishing social responsibility, a sub-factor of CSM. On the other hand, brand trust did not affect economic viability and environmental soundness, which are also both sub-factors of sustainable management. According to the result, it can also be assumed that consumers think that a company’s investment in product development, quality improvement, R&D investment, and service efforts is not because consumers trust the brand, but because it is logical. In addition, if a company releases a product using a trusted brand, it is more likely to succeed than otherwise, and thus consumers might think that the company does not strive for eco-friendliness or improvements in the lives of local residents. For that reason, companies need to put in their efforts in further strengthening brand trust in order to improve CSM, which fulfills the responsibilities and obligations as members of society, as well as corporate image.

The outcomes of this study present theoretical implications that can be applied to the rapidly changing business environment. Through this study, it became clear that establishing compatible and authentic CSR as well as brand trust is essential for sustainable development. On that account, this study is significant as it confirmed that CSR activities suitable for a company do not solely spend money. Instead, they strengthen corporate and brand trust and enable a company to engage in sustainable management that helps humans and the environment.

Unlike previous studies, this study drew results on strategic planning and operation of CSM from the perspective of consumers. In other words, although CSR was mostly used as an outcome variable based on strategy, this study contributed to the development of previous studies related to CSR and CSM by deducing the results that consumer perceived CSR enhances brand trust and brand trust improves CSM. In addition, although cosmetic companies actively engage in CSR for sustainable management, research on this has been insufficient. Accordingly, this study suggested the strategic and policy direction for cosmetic companies to pursue sustainable management by examining the relationship between CSR, brand trust, and CSM.

Although this study focused on presenting significant results and implications, there are problems that need to be dealt with in the future. The limitations of this study are as follows. First, since this study targets cosmetics companies, the results cannot be applied to other companies in various industries. Hence, future studies must examine compatibility and authenticity of CSR and the factors of brand trust in various industries. Second, since this study used questionnaires reported by individuals, common method bias may have occurred in conformity with individual characteristics. Thus, the quality of research method must be improved by incorporating qualitative methods such as observation and indepth interviews. Lastly, this study only looked into the main and mediated effects. Therefore, different moderating variables that can be used for various multiple group analyses have to be considered and reapplied to this research model.

## Data Availability Statement

The original contributions presented in the study are included in the article/supplementary material, further inquiries can be directed to the corresponding author.

## Author Contributions

G-YJ contributed to the derivation of ideas, practical analysis, and wrote conclusions and recommendations of the thesis. S-HL contributed to the theoretical background and hypothesis setting and data collection. Both authors contributed to the article and approved the submitted version.

## Conflict of Interest

The authors declare that the research was conducted in the absence of any commercial or financial relationships that could be construed as a potential conflict of interest.

## Publisher’s Note

All claims expressed in this article are solely those of the authors and do not necessarily represent those of their affiliated organizations, or those of the publisher, the editors and the reviewers. Any product that may be evaluated in this article, or claim that may be made by its manufacturer, is not guaranteed or endorsed by the publisher.

## Appendix

**APPENDIX TABLE 1:** Measurement item.

Construct		Measurement Item	Number	Measure	References
CSR	Compatibility	CSR is highly relevant to a company’s business	3	Five-Point Likert scale	Becker-Olsen and Hill, 2006; O’Connor and Meister, 2008
		CSR is highly associated with corporate image			
		CSR is highly relevant to target customers			
	Authenticity	CSR has sincerity that it is for the public good	3	Five-Point Likert scale	Alhouti et al., 2016
		CSR contributes society			
		CSR truly care for social abbreviations			
Brand trust		Corporation has professional skills	5	Five-Point Likert scale	Keller and Aaker, 1992; Mayer et al., 1995
		Corporation has the ability to develop products that meet the needs of consumers			
		Corporation is striving for mutual benefit with consumers			
		Corporation is trying to provide consumers with the right information			
		Corporation is striving to accept consumer requirements correctly			
CSM	Economic viability	Corporation is striving to improve the quality of the product	4	Five-Point Likert scale	Amalric and Hauser, 2005
		Corporation is working to improve sales			
		Corporation is striving to improve profits			
		Corporation is striving to develop core technologies to create future values			
	Environmental soundness	Corporation is working to improve the environment	4	Five-Point Likert scale	Chen et al., 2006
		Corporation is working to protect the environment			
		Corporation is striving to develop eco-friendly products			
		Corporation is working hard to ensure that their products are recycled			
	Social responsibility	Corporation is striving to contribute to social development	4	Five-Point Likert scale	Kotler and Lee, 2005
		Corporation is actively engaged in donation activities			
		Corporation contributes to stabilizing society			
		Corporation is making efforts to create jobs and develop local communities			
